# Preventive use of beta-blockers for anthracycline-induced cardiotoxicity: A network meta-analysis

**DOI:** 10.3389/fcvm.2022.968534

**Published:** 2022-08-11

**Authors:** Dongsheng He, Jun Hu, Ying Li, Xiaofei Zeng

**Affiliations:** ^1^Department of Cardiothoracic Surgery, The First Affiliated Hospital of Chengdu Medical College, Chengdu, China; ^2^Department of Cardiology, The First Affiliated Hospital of Chengdu Medical College, Chengdu, China

**Keywords:** beta-blockers, anthracycline, cardiotoxicity, systematic review, network meta-analysis

## Abstract

**Background:**

Anthracyclines are commonly used chemotherapeutic agents to treat malignant tumors. However, cardiotoxicity is a potentially serious adverse effect of anthracyclines. Beta-blockers may be effective in preventing anthracycline-induced cardiotoxicity (AIC). However, the lack of direct comparisons of various beta-blockers interferes with clinical decision-making. Network meta-analysis (NMA) was performed to assess the effectiveness of beta-blockers for AIC.

**Methods:**

We searched PubMed, Embase, Web of Science, and the Cochrane Central Register of Clinical Trials. The last update was in May 2022. Randomized controlled trials (RCT) of beta-blockers for AIC were included. Four beta-blockers were selected for comparison based on the number of studies. NMA was conducted with STATA 14.0 software.

**Results:**

A total of 10 RCTs (875 patients) met the selection criteria. NMA results showed that carvedilol was superior to bisoprolol [*SMD* = −0.50, 95% *CI* (−0.91, −0.10)] and nebivolol [*SMD* = −1.46, 95%*CI* (−2.82, −0.11)] in a delay of LVEF. The results of the cumulative probability ordering are as follows: carvedilol (83.8%) > metoprolol (71.8%) > bisoprolol (43.9%) > placebo (40.9%) > nebivolol (9.5%).

**Conclusion:**

Based on the available evidence, carvedilol is the best beta-blocker for AIC, followed by metoprolol. However, additional studies with large samples should be conducted to confirm our findings.

## Introduction

Anthracyclines are anticancer drugs, including Adriamycin, erythromycin, and epi-amycin. They can be used to treat various types of cancer, including breast cancer, lymphoma, and leukemia (1, 2). Although their anticancer effects are notable, numerous clinical studies have found these drugs have serious adverse effects, with cardiotoxicity particularly prominent (3, 4). Anthracycline-induced cardiotoxicity (AIC) is a dose-limiting and possibly fatal complication of anthracycline administration that can arise during any period of chemotherapy (5). The main representative features are arrhythmias, pericardial effusion, and myocardial ischemia. AIC can contribute to cardiac failure and decrease survival (6). Mechanisms of AIC are complex and include free radicals, calcium overload, impaired energy metabolism, and apoptosis (7–12). A liposome-encapsulated formulation, doxorubicin liposome, was developed to limit anthracycline exposure in the myocardium. Liposomal doxorubicin improves the therapeutic index of conventional anthracyclines (13). However, AIC is still a pressing issue.

Beta-blockers are effective in treating hypertension, heart disease, and cardiac failure (14–16). Beta-blockers improve ventricular remodeling and reduce arrhythmias mainly by altering the status of adrenergic receptors (17, 18). Beta-blockers can delay the progression to heart failure in patients who develop cardiotoxicity (19, 20). Several beta-blockers are already available for clinical use, such as metoprolol, atenolol, nebivolol, bisoprolol, and carvedilol. Studies have shown that beta-blockers could be effective in preventing AIC (21). However, previous meta-analyses that evaluated the efficacy of beta-blockers to ameliorate AIC in terms of changes in left ventricular ejection function (LVEF) showed inconsistent results (19, 22–26). Furthermore, due to the small sample size of most studies, there is a lack of direct comparison between beta-blockers to determine which beta-blocker is most effective in preventing AIC.

Network meta-analysis (NMA) synthesizes evidence from direct and indirect comparisons to rank treatment interventions and guides drug selection (27). To provide additional evidence on beta-blocker treatment against AIC, a comprehensive systematic evaluation and NMA of relevant randomized controlled trials (RCTs) were conducted to assess which beta-blockers provided the best cardioprotective effect.

## Methods

Network meta-analysis was conducted following the guidelines of the Preferred Reporting Items for Systematic Reviews and Meta-Analysis Statements for Network Meta-Analysis (PRISMA-NMA) (28).

### Inclusion and exclusion criteria

The inclusion criteria were: (1) participants: patients were diagnosed with tumors by pathology or imaging and were older than 18 years. All patients received anthracyclines, including epirubicin, pirarubicin, or doxorubicin. The dose and duration of drug treatment were unlimited; (2) type of study: randomized clinical trials (RCTs); (3) interventions: the experimental group began using beta-blockers before chemotherapy to counteract the cardiotoxicity of anthracycline-based chemotherapy. In the control group, a placebo was used; and (4) outcomes: LVEF at baseline and after chemotherapy, mortality, and adverse events. The exclusion criteria were: (1) non-RCT; (2) studies with insufficient data, duplicate data, or data that could not be extracted; (3) cardiotoxicity due to non-anthracycline chemotherapy; and (4) reviews, conference abstracts, or meta-analysis.

### Search strategy

Randomized clinical trial studies on beta-blockers for the prevention of AIC were searched in PubMed, Embase, Web of Science, and the Cochrane Central Register of Clinical Trials. The last search was in May 2022. References from included studies were checked to identify additional studies. Search terms included anthracycline, chemotherapy, cardiotoxicity, doxorubicin, atenolol, carvedilol, metoprolol, nebivolol, bisoprolol, arotinolol, adrenergic beta-antagonists, randomized controlled trial, and random^*^. The search was conducted using a combination of medical subject headings and free-text words (Supplementary Table S1).

### Data extraction

Data extracted included: (1) basic information: title, source, author, year; (2) baseline characteristics of the study population: number of trial participants, age, disease type; (3) intervention details, follow-up time; (4) key elements of the risk of bias evaluation; and (5) data on outcome indicators and outcome measures: LVEF, adverse events, and mortality.

### Quality assessment

Two investigators independently assessed the risk of bias in the included studies and cross-checked the results. A bias assessment tool recommended in the Cochrane Handbook 5.1.0 was used to evaluate the risk of bias in RCT (29).

### Statistical analysis

Stata 14.0 was used to analyze the data. Standardized mean difference (*SMD*) was used as the effect analysis statistic for the measurement data. Dichotomous variables were analyzed using the risk ratio (*RR*) as the effect analysis statistic. Each effect size was provided with its 95% confidence interval (95%*CI*). The χ^2^ test was used to assess statistical heterogeneity between the results of the studies, while the magnitude of heterogeneity was determined by combining *I*^2^ quantification. Fixed effects were used if there was no heterogeneity between studies (*I*^2^ <50%, *P* > 0.1). If there was heterogeneity (*I*^2^ > 50%, *P* < 0.1), the source of heterogeneity was analyzed, and a meta-analysis was performed with random effects after excluding the influence of heterogeneity.

Due to the limited data available in the literature, subgroup analyses were only performed for doses of carvedilol (6.25 mg vs. 12.5 mg vs. 25 mg). Sensitivity analysis was used to test the stability of the meta-analysis results. The mvmeta package was used for data preprocessing in the NMA. When network relationship diagrams were drawn, inconsistency tests should be performed to determine if there were closed loops in the network relationship diagrams. In the present study, no closed loop was formed for each outcome indicator. Therefore, no inconsistency test was performed. The outcome indicators for each intervention were ranked by plotting the surface under the cumulative ranking curve (SUCRA). Comparison-adjusted funnel plots were used to assess publication bias and the effects of the small sample in included studies.

## Results

### Search results

**Figure 1:**
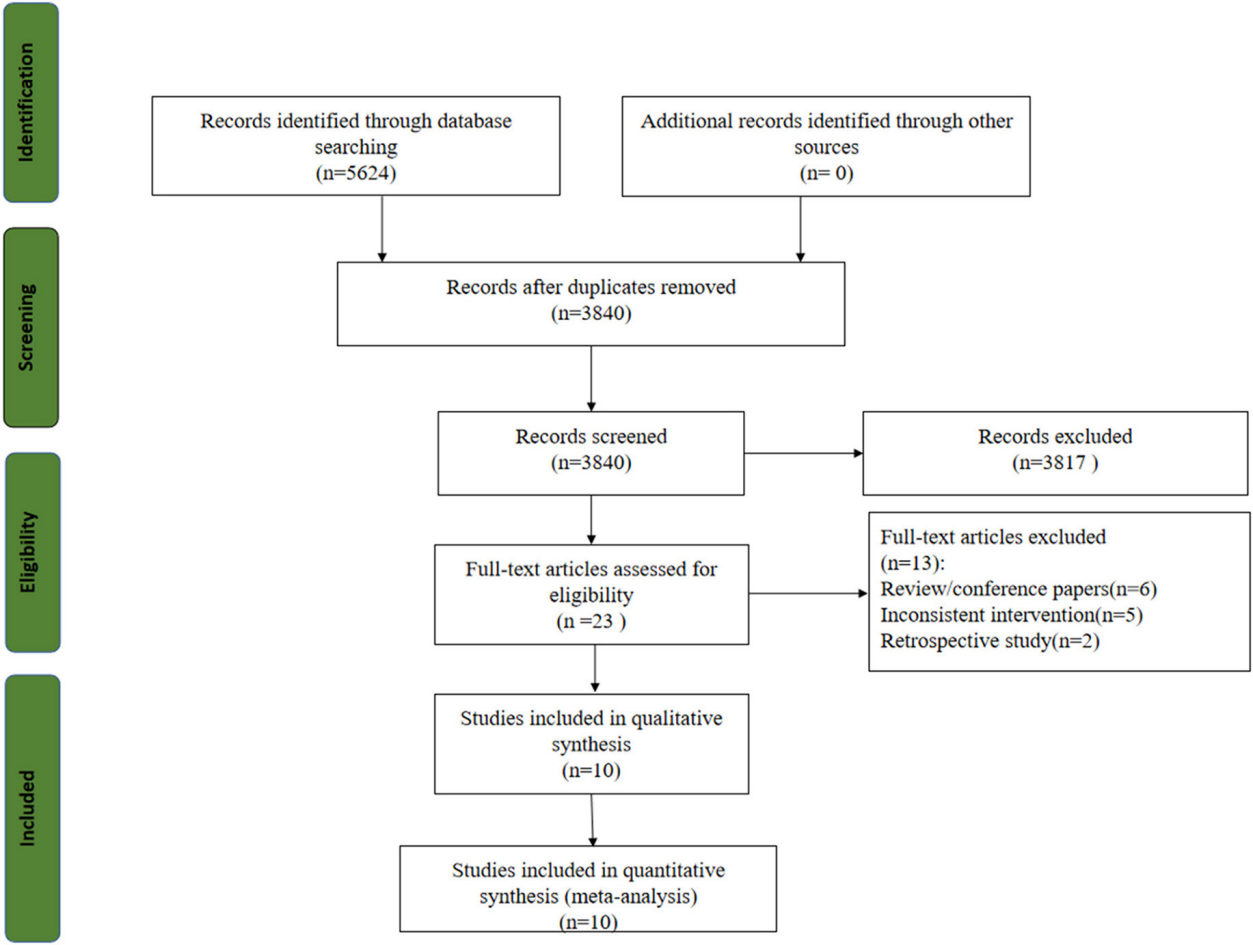
Flow chart of article screening process.

Five thousand six hundred twenty-four studies were obtained from the initial review, and 10 RCTs were included after screening (21, 30–38). The study selection flow chart is shown in Figure 1.

### Study and patient characteristics

There were 875 patients in the 10 RCTs. Most of the tumor types were breast cancer. Four beta-blockers were included: bisoprolol, metoprolol, nebivolol, and carvedilol. Two studies compared the efficacy of different doses of beta-blockers compared to placebo. The beta-blocker doses ranged from 5 to 100 mg. The experimental groups were comparable to the control groups at baseline in these RCTs. The characteristics of the included studies are shown in Table 1.

**Table 1:** General characteristics of selected RCTs.

**Author year**	**State**	**Mean age**		**Sample size**		**Cancer type**	**Drug type**	**Doses**	**Follow-up duration**	**Outcome indicators**
		**T**	**C**	**T**	**C**					
Livi et al., 2021 (36)	Italy	24~75	24~75	45	42	Breast cancer	Bisoprolol	5 mg	24, m	LVEF
Gulati et al., 2016 (33)	Norway	50.5 ± 9.1	50.8 ± 9.2	30	30	Breast cancer	Metoprolol	100 mg	NA	LVEF, side effects
Kaya et al., 2013 (35)	Turkey	51.4 ± 9.4	50.5 ± 11.1	27	18	Breast cancer	Nebivolol	5 mg	NA	LVEF
Abuosa et al., 2018 (30)	Saudi Arabia	46.1 ± 13.0/41.3 ± 18.2/ 42.0 ± 15.0	40.4 ± 14.0	41/38/37	38	Mostly breast cancer	Carvedilol	6.25 mg/12.5 mg/25 mg	6, m	LVEF, mortality
Avila et al., 2018 (31)	Brazil	50.8 ± 10.10	52.9 ± 9.05	96	96	Breast cancer	Carvedilol	25 mg	6, m	LVEF, mortality, side effects
Beheshti et al., 2016 (38)	Iran	29~54	29~54	30	40	Breast cancer	Carvedilol	6.25 mg	NA	LVEF
Elitok et al., 2014 (32)	Turkey	33.4 ± 5.8	34.3 ± 6.1	40	40	Breast cancer	Carvedilol	12.5 mg	6, m	LVEF
Kalay et al., 2006 (34)	Turkey	46.8 ± 14	49.0 ± 9.8	25	25	Mostly breast cancer	Carvedilol	12.5 mg	6, m	LVEF, mortality
Nabati et al., 2017 (37)	Iran	47.57 ± 8.75	47.1 ± 12.17	46	45	Breast cancer	Carvedilol	12.5 mg	6, m	LVEF
Salehi et al., 2011 (21)	Iran	45.70 ± 14.16/ 52.52 ± 11.00	43.50 ± 15.27	15/17	14	Breast cancer and lymphoma	Carvedilol	12.5 mg/25 mg	4, m	LVEF

*NA, data not available; T, treatment group; C, control group; m, months; LVEF, left ventricular ejection fraction*.

### Quality assessment results

**Figure 2:**
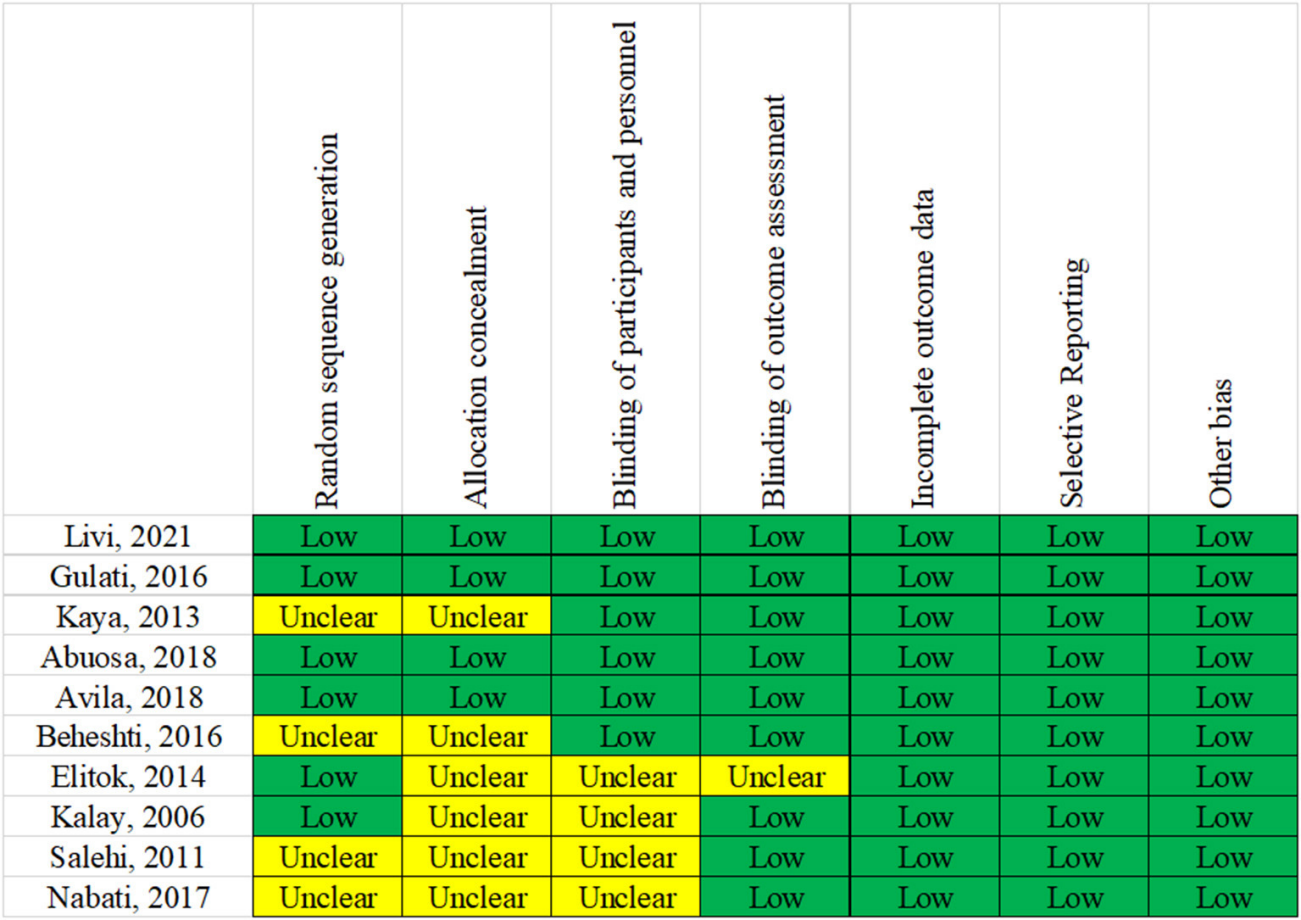
Risk of bias in the included studies.

All included RCTs mentioned that the grouping was performed using a random method. Bias risk assessment for randomization showed low-risk bias in six RCTs and unclear in four RCTs. Regarding the concealment of random assignment, four RCTs showed low-risk bias, and six showed unclear results. Placebos were used in all RCTs to implement the blinded method. Regarding data completeness, selective reporting and other aspects showed a low risk of bias. The bias evaluation is shown in Figure 2.

### Pairwise comparison of meta-analysis results

**Figure 3:**
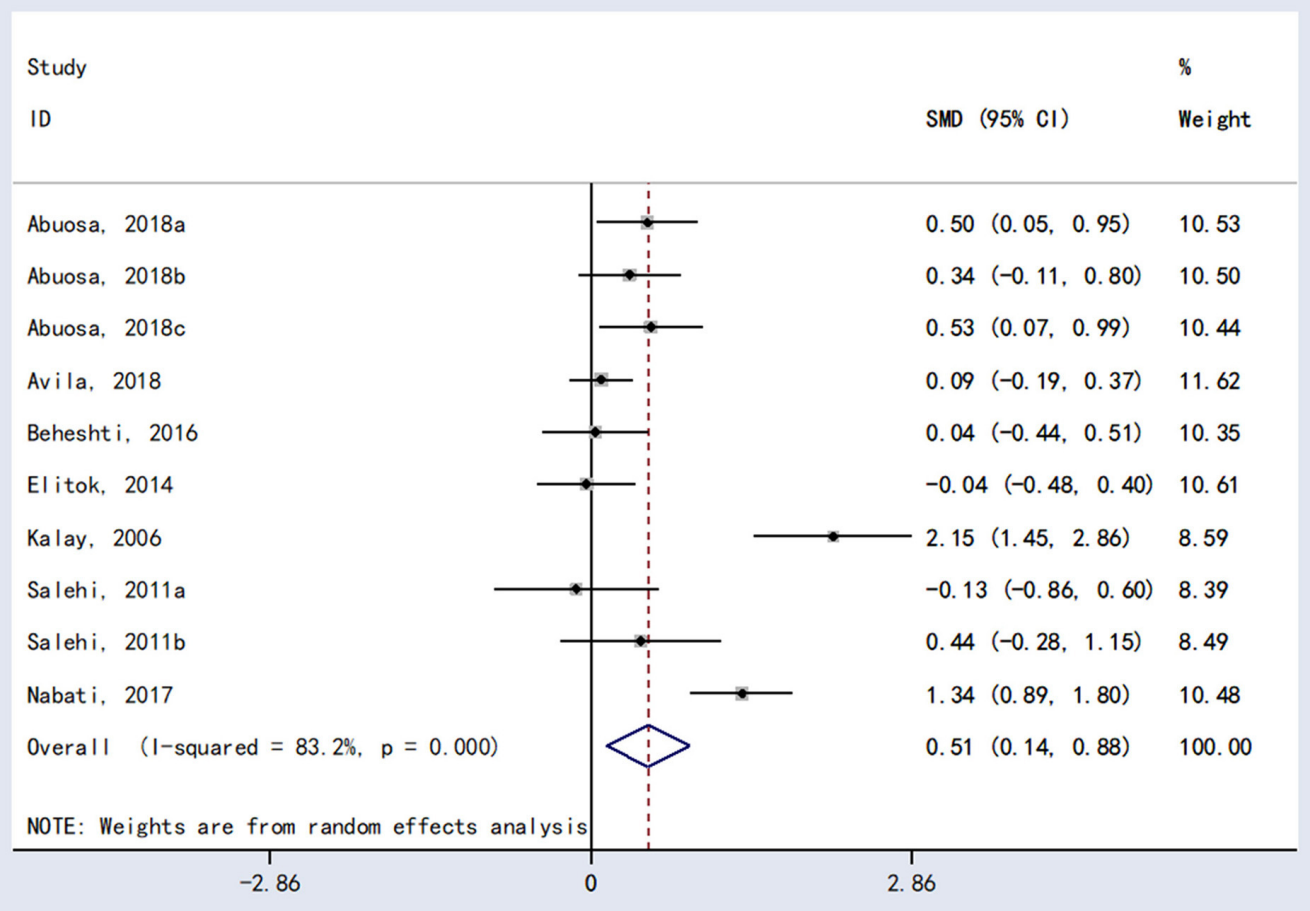
The forest plot of the effect of carvedilol on LVEF.

The meta-analysis showed a statistically significant difference between carvedilol and placebo in LVEF [*RR* = 0.51, 95%*CI* (0.14, 0.88), *P* = 0.007; Figure 3].

Compared to placebo, bisoprolol and nebivolol had an advantage in LVEF, with a statistically significant difference [*RR* = 0.67, 95%*CI* (0.23, 1.10), *P* = 0.002; *RR* = 1.49, 95% *CI* (0.82, 2.16), *P* < 0.0001]. However, the difference between metoprolol and placebo was not statistically significant [*RR* = 0.06, 95%*CI* (−0.44, 0.57), *P* = 0.803].

**Figure 4:**
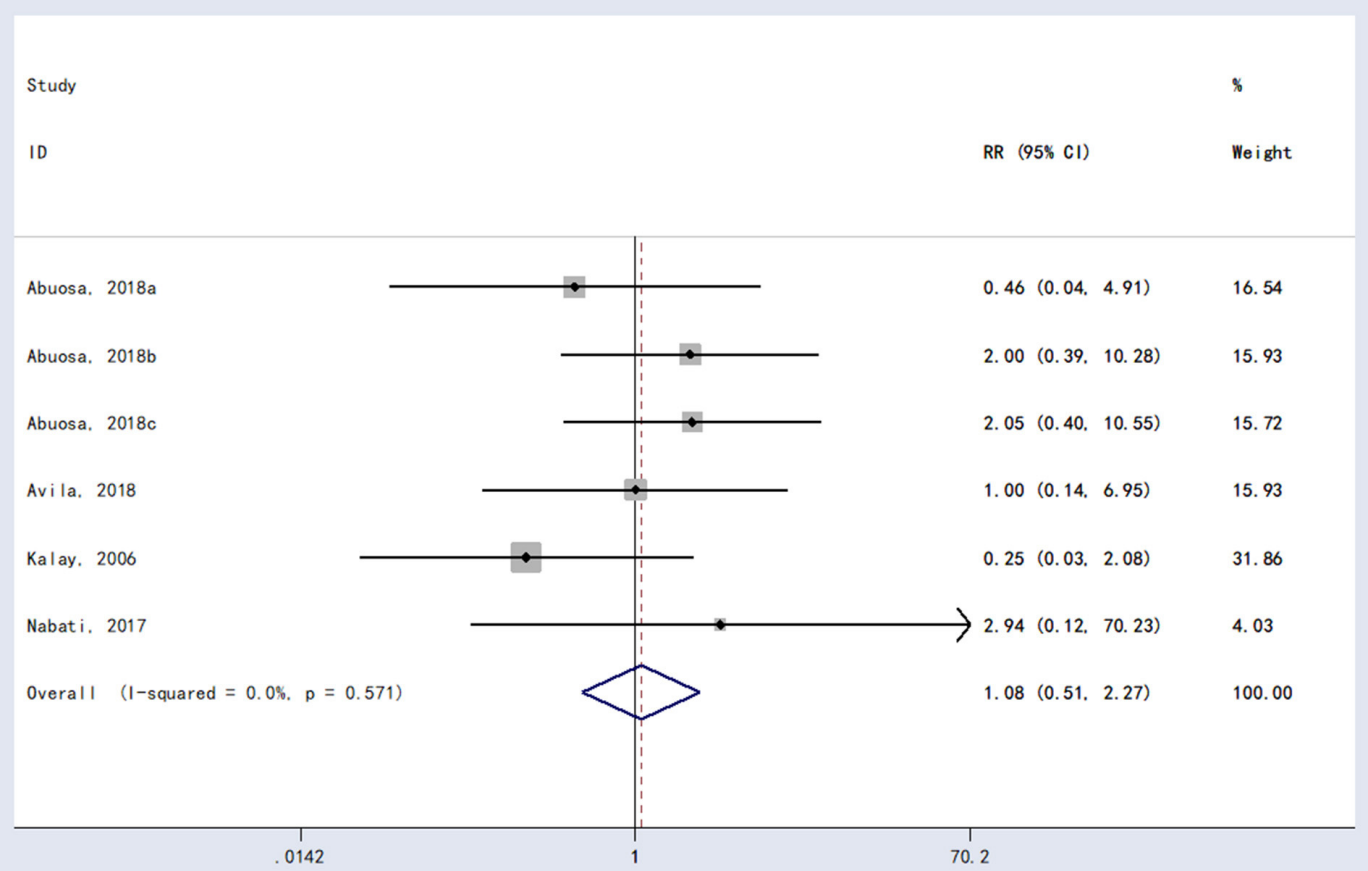
Forest plot of Carvedilol's impact on mortality.

In terms of mortality, the meta-analysis did not show statistically significant differences between carvedilol and placebo (*RR* = 1.08, 95% *CI* (0.51, 2.27), *P* = 0.889; Figure 4].

### Adverse events

There were no significant differences in adverse events between metoprolol and placebo [*RR* = 4.00, 95% *CI* (0.47, 33.73), *P* = 0.203] or carvedilol and placebo [*RR* = 0.50, 95% *CI* (0.13, 1.94), *P* = 0.317].

### Subgroup analysis

**Figure 5:**
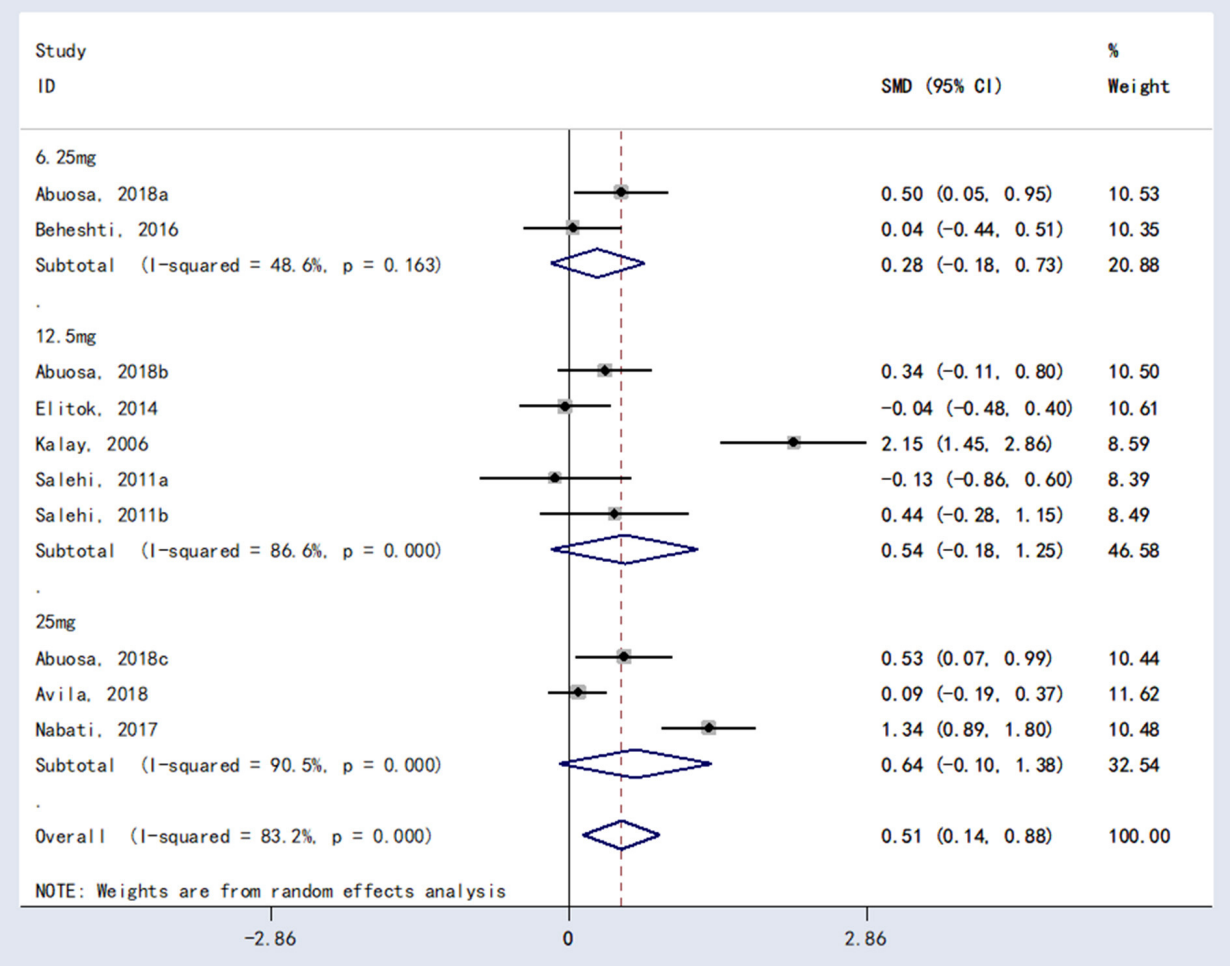
Subgroup analysis forest plot of effects of different doses of carvedilol on LVEF.

Subgroup analysis was performed for different doses of carvedilol (6.25 mg vs. 12.5 mg vs. 25 mg). There were no statistically significant differences in LVEF performance between 6.25 and 12.5 mg [*RR* = 0.28, 95%*CI* (−0.18, 0.73), *P* = 0.234] or between carvedilol 6.25 mg carvedilol and 25 mg [*RR* = 0.54, 95%*CI* (−0.18, 1.25), *P* = 0.140] or between carvedilol 12.5 mg carvedilol and 25 mg [*RR* = 0.64, 95%*CI* (−0.10, 1.38), *P* = 0.091]. The results are shown in Figure 5.

### Sensitivity analysis

**Figure 6:**
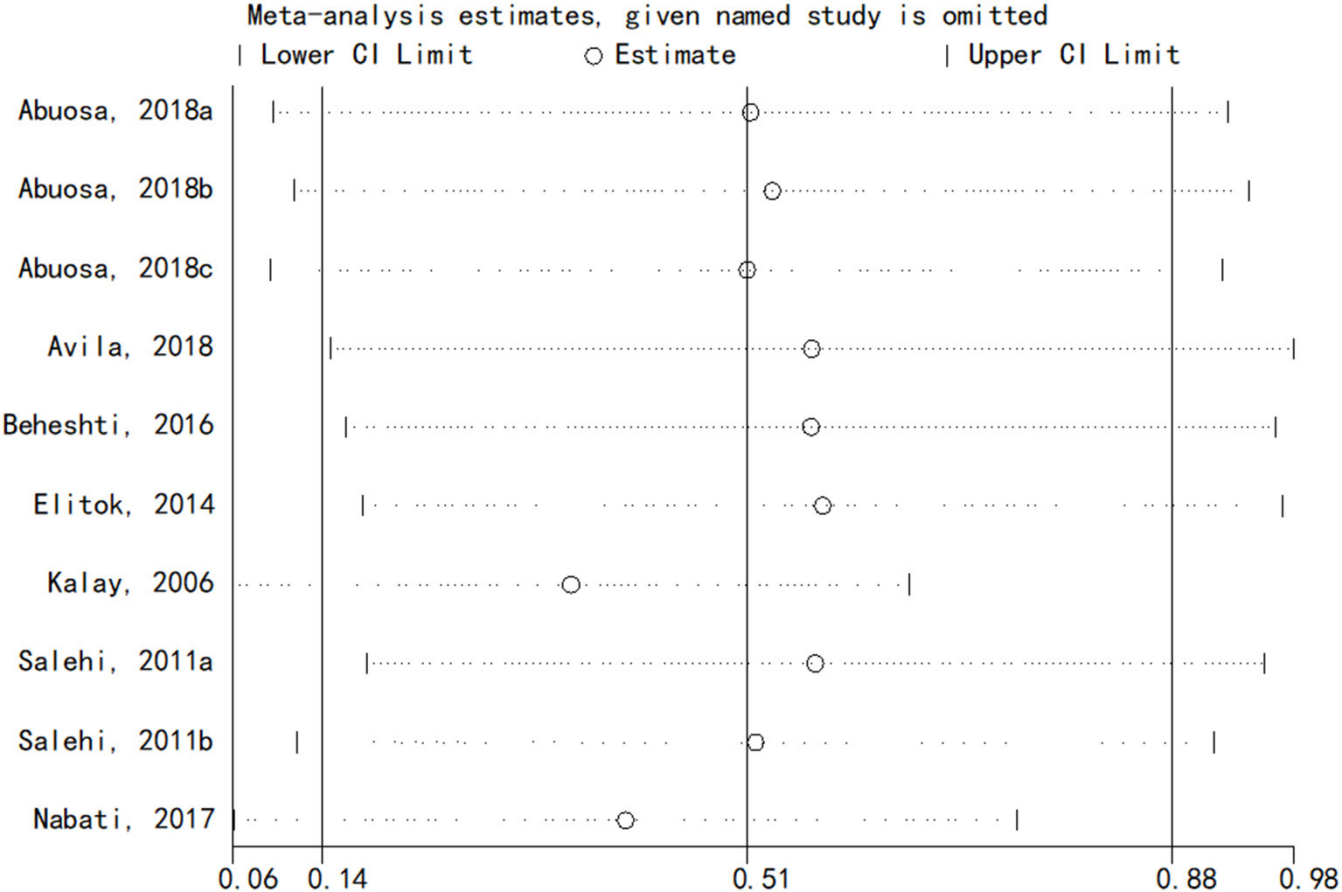
Sensitivity analysis results.

The sensitivity analysis of the results of LVEF of carvedilol compared to placebo was performed using the one-by-one elimination method. The results showed that the meta-analysis results were stable (Figure 6).

### Results of network meta-analysis

**Figure 7:**
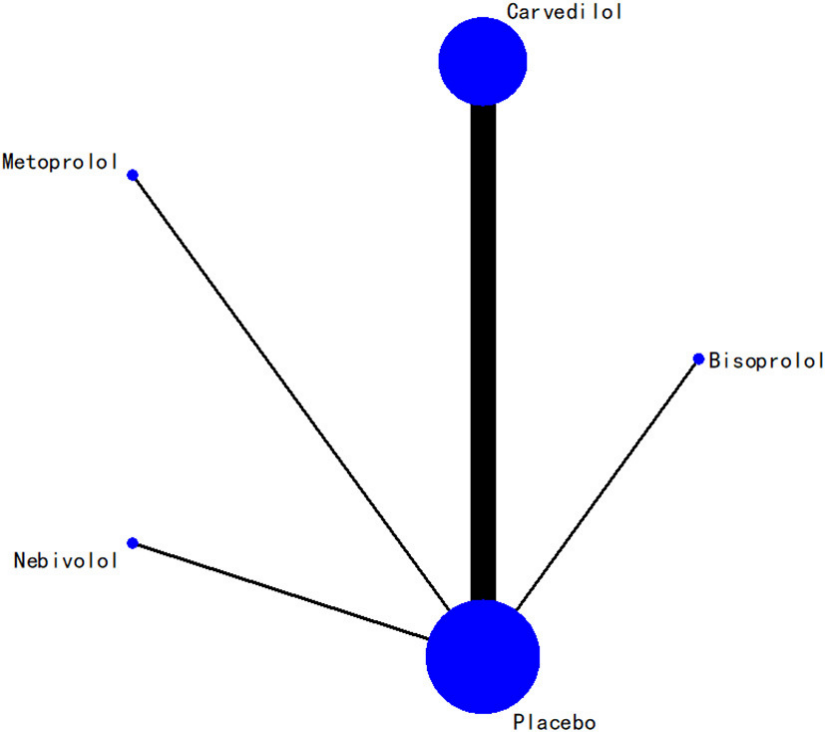
Evidence network diagram.

In terms of LVEF, the network relationships for the four beta-blockers are shown in Figure 7. According to the evidence network diagram of NMA comparisons, the width of each edge is proportional to the number of RCTs comparing each pair of treatments, and the size of each treatment node is proportional to the number of randomized participants (sample size).

**Figure 8:**
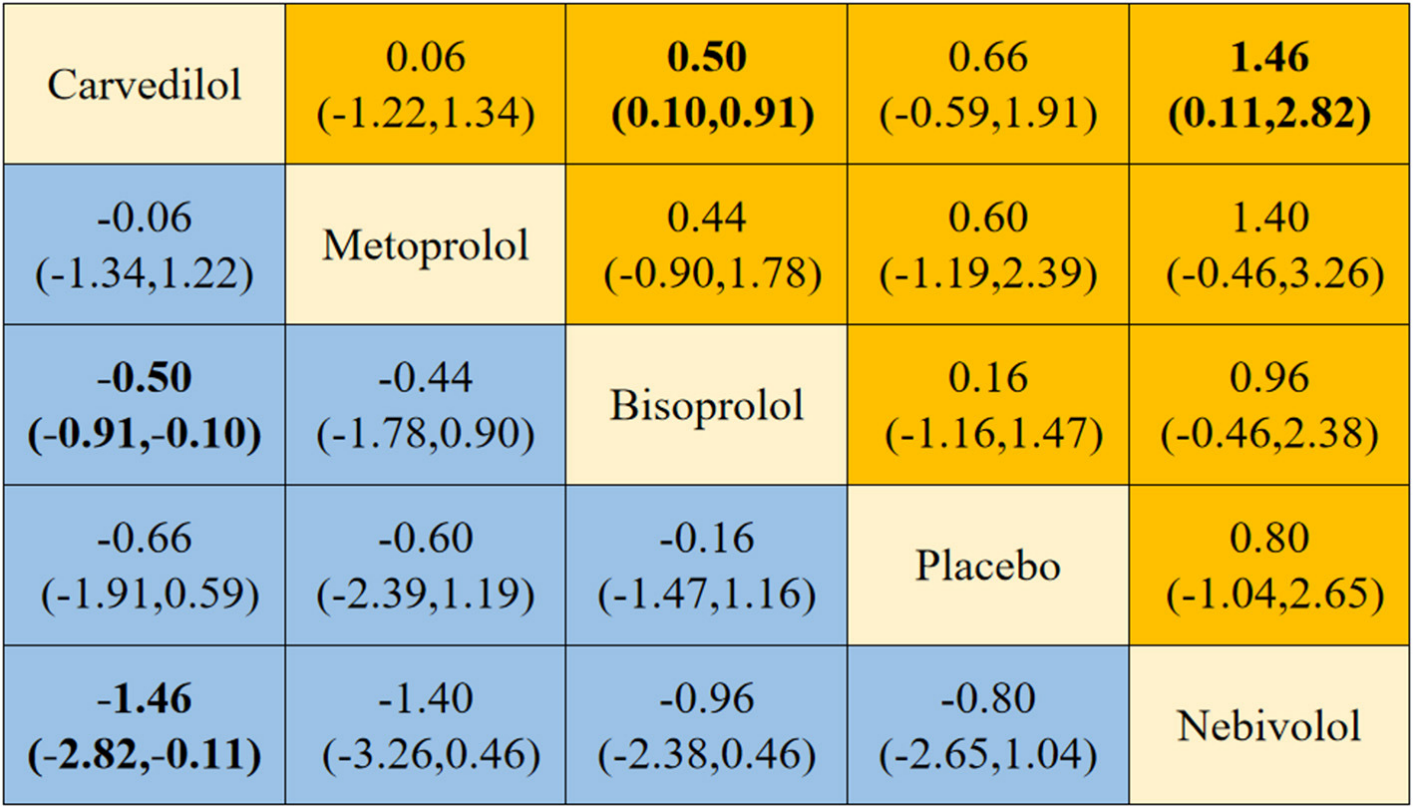
Results of network meta-analysis.

Four different beta-blockers were subjected to NMA, yielding 10 two-by-two comparisons, two of which were statistically significant (Figure 8). NMA results showed that carvedilol was superior to bisoprolol [*SMD* = −0.50, 95% *CI* (−0.91, −0.10)] and nebivolol [*SMD* = −1.46, 95% *CI* (−2.82, −0.11)] in delaying the reduction in LVEF.

### Probability ranking result

**Figure 9:**
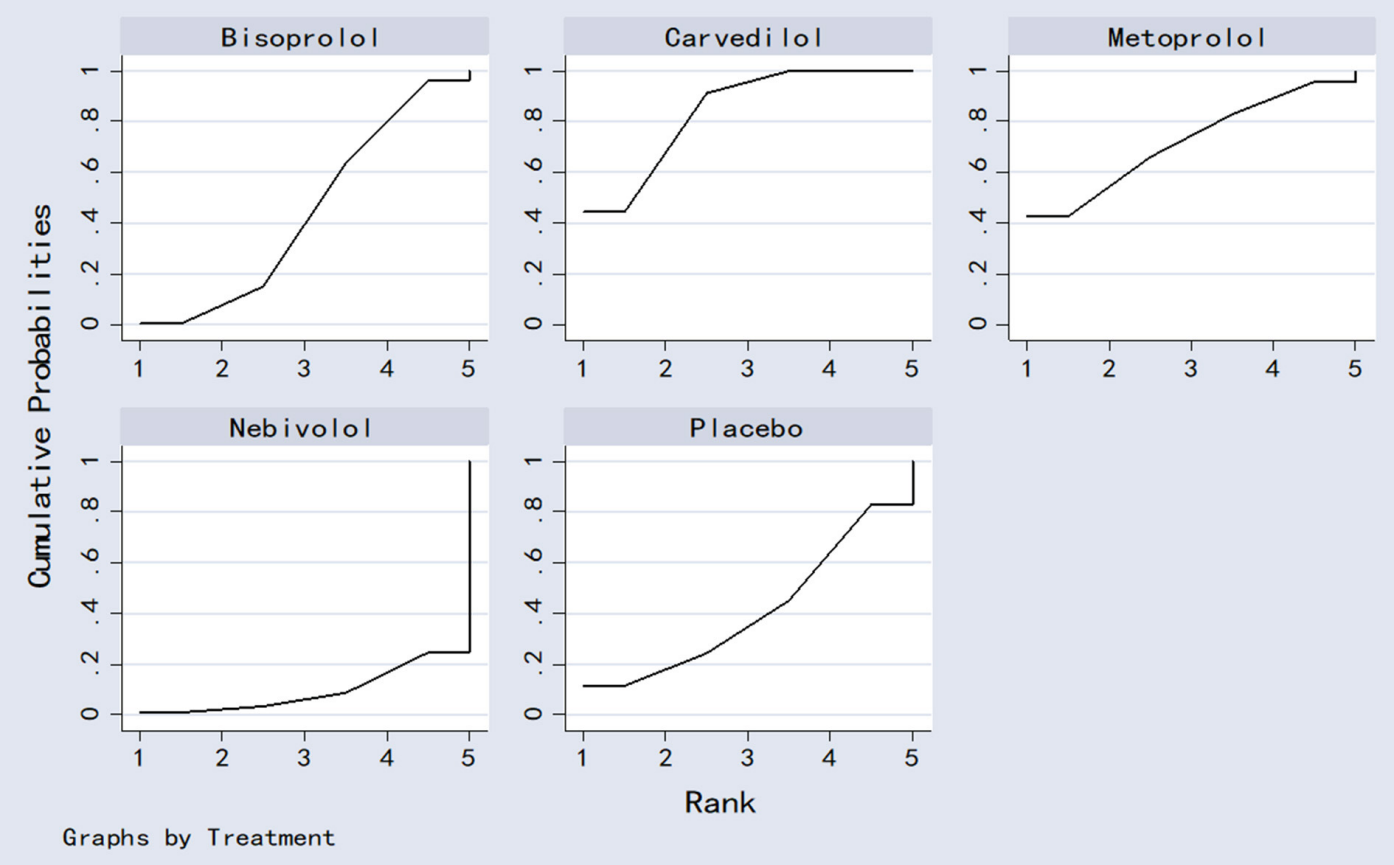
The surface under the cumulative ranking curve plots.

The four beta-blockers were ranked based on the SUCRA values (Figure 9). The results of the cumulative probability ordering are as follows: carvedilol (83.8%) > metoprolol (71.8%) > bisoprolol (43.9%) > placebo (40.9%) > nebivolol (9.5%).

### Publication bias analysis

**Figure 10:**
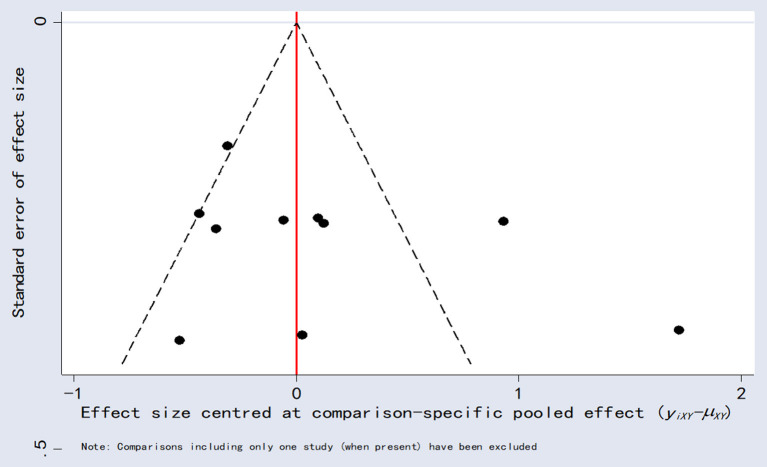
Comparison-correction funnel plot.

Comparison-adjusted funnel plots for LVEF as an outcome indicator were plotted for publication bias. These results showed poor symmetry, suggesting a possible degree of publication bias (Figure 10).

## Discussion

This study evaluated the efficacy of different beta-blockers vs. placebo for preventing AIC by NMA. NMA included 10 RCTs, including 875 patients. Beta-blockers included in the NMA were bisoprolol, metoprolol, nebivolol, and carvedilol. A previous meta-analysis confirmed the carvedilol cardioprotective effects in patients treated with anthracyclines, improving the significant decrease in LVEF and reducing the incidence of cardiovascular events (23). Long-term follow-up studies have shown that LVEF at the end of treatment is an independent predictor of cardiotoxicity (39). Lower LVEF is associated with an increased risk of cardiotoxicity (40). Therefore, a decrease in LVEF is recommended to define chemotherapy-related cardiotoxicity (41, 42).

Anthracyclines are among the most popular chemotherapy drugs due to their broad-spectrum and potent anticancer effects (43). While it provides sound anticancer therapeutic effects, the development of cardiotoxicity limits its application. The cardiotoxic effects become more pronounced as the cumulative dose increases. The risk of congestive heart failure is positively correlated with anthracycline doses (44–46). Early monitoring and timely intervention are essential to avoid the progression to irreversible heart damage (47). Beta-blockers can treat heart failure by stimulating the Gs-AC-cAMP-PKA signaling pathway to produce positive inotropic effects in cardiac myocytes (48).

The results of the direct comparative meta-analysis of this study showed an advantage of carvedilol in causing the delay in the reduction of LVEF compared to placebo. The results are consistent with other studies (22, 23, 25). The result stability was also confirmed by sensitivity analysis ruling out the possibility of false-positive results. In addition, bisoprolol and nebivolol were equally advantageous in mitigating the decline in LVEF. However, more evidence is needed to support the findings due to the size of the included studies. Unlike placebo, metoprolol was not statistically significant in mitigating the LVEF decline. This may be related to the ineffective protective effect against cardiotoxicity due to the absence of antioxidant activity of metoprolol (49).

The NMA results showed that carvedilol was superior to bisoprolol and nebivolol in delaying LVEF reduction. The results of the probability ranking indicated that carvedilol was the best beta-blocker to prevent AIC. Based on direct comparisons, carvedilol and placebo had no statistically significant difference in mortality. Therefore, we recommend carvedilol as the preferred regimen for preventing AIC. Carvedilol is an antioxidant and has more potent antioxidant properties than other types of beta-blockers (34). The metabolites of carvedilol exhibit antioxidant properties. The metabolites are 50 or 100 times more powerful than carvedilol (50). Carvedilol inhibits the lipid peroxidation in cardiac cell membranes and oxygen release from neutrophils. It preserves the body's natural antioxidant system by scavenging peroxides, hypochlorous radicals, and oxygen radicals (51). We attempted to compare the effect of different doses of carvedilol in delaying the reduction of LVEF by subgroup analysis. Unfortunately, no meaningful recommended dose was found. Therefore, future studies with varying doses of carvedilol to prevent AIC should be conducted.

## Limitations

First, because of the lack of direct comparisons between different beta-blockers in included studies, the comparisons between other beta-blockers in NMA were obtained by indirect comparisons. Therefore, the results, effectiveness, and safety of the actual drugs may be biased. Second, the included studies were mainly focused on carvedilol (seven studies), while there was only one study for bisoprolol, metoprolol, and nebivolol. Therefore, the results of the studies were prone to bias. Finally, the small sample size of patients included in some of the studies may reduce the credibility of the trial results.

## Conclusions

Carvedilol may be the best beta-blocker for preventing AIC, followed by metoprolol. To confirm and support the findings of this NMA, larger sample sizes and high-quality RCTs are needed.

## Data availability statement

The original contributions presented in the study are included in the article/Supplementary material, further inquiries can be directed to the corresponding authors.

## Author contributions

DH and JH write this paper and analyze the data. YL and XZ design this study, perform the statistical analysis, and review this paper. All authors contributed to the article and approved the submitted version.

## Funding

This study was supported by Scientific Research Project of Chengdu Municipal Health Commission (No. 2021066) and Sichuan Medical Association (Youth Innovation) Scientific Research Project (Q21067).

## Conflict of interest

The authors declare that the research was conducted in the absence of any commercial or financial relationships that could be construed as a potential conflict of interest.

## Publisher's note

All claims expressed in this article are solely those of the authors and do not necessarily represent those of their affiliated organizations, or those of the publisher, the editors and the reviewers. Any product that may be evaluated in this article, or claim that may be made by its manufacturer, is not guaranteed or endorsed by the publisher.
