# Supplementary material for: Preventive use of beta-blockers for anthracycline-induced cardiotoxicity: A network meta-analysis
Source: Front Cardiovasc Med. 2022 Aug 11;9:968534. doi: 10.3389/fcvm.2022.968534 (PMC9403514; doi:10.3389/fcvm.2022.968534)
Supplement: Supplementary file 1 [file Table_1.DOCX]

| Table S1.Search strategy(“Pubmed”) | |
| --- | --- |
| Number | Search terms |
| #1 | Anthracyclines[Mesh] |
| #2 | Anthracyclin*[Title/Abstract] |
| #3 | Aclarubicin[Title/Abstract] |
| #4 | aclacinomycin [Title/Abstract] |
| #5 | Aldoxorubicin[Title/Abstract] |
| #6 | Annamycin[Title/Abstract] |
| #7 | Daunorubicin[Title/Abstract] |
| #8 | Cerubidine[Title/Abstract] |
| #9 | Doxorubicin[Title/Abstract] |
| #10 | Adriamycin[Title/Abstract] |
| #11 | Epirubicin [Title/Abstract] |
| #12 | GPX-150[Title/Abstract] |
| #13 | Idarubicin[Title/Abstract] |
| #14 | Pirarubicin [Title/Abstract] |
| #15 | Plicamycin[Title/Abstract] |
| #16 | Mithracin [Title/Abstract] |
| #17 | Sabarubicin [Title/Abstract] |
| #18 | SP1049C[Title/Abstract] |
| #19 | Valrubicin[Title/Abstract] |
| #20 | Valstar[Title/Abstract] |
| #21 | Zoptarelin doxorubicin[Title/Abstract] |
| #22 | Zorubicin[Title/Abstract] |
| #23 | LHRH,lysine(6)-doxorubicin[Title/Abstract] |
| #24 | Plicamycin[Mesh] |
| #25 | Idarubicin[Mesh] |
| #26 | Epirubicin[Mesh] |
| #27 | Doxorubicin[Mesh] |
| #28 | Daunorubicin[Mesh] |
| #29 | Aclarubicin[Mesh] |
| #30 | or/1-30 |
| #31 | Adrenergic beta-Antagonists[Mesh] |
| #32 | Beta blocker*[Title/Abstract] |
| #33 | Beta-adrenergic blocking agents[Title/Abstract] |
| #34 | Beta-adrenergic antagonists[Title/Abstract] |
| #35 | Beta adrenergic receptor antagonists[Title/Abstract] |
| #36 | Bisoprolol[Mesh] |
| #37 | Bisoprolol[Title/Abstract] |
| #38 | Cardicor[Title/Abstract] |
| #39 | Emcor[Title/Abstract] |
| #40 | Carvedilol[Mesh] |
| #41 | Carvedilol[Title/Abstract] |
| #42 | Coreg [Title/Abstract] |
| #43 | Atenolol[Mesh] |
| #44 | Tenormin [Title/Abstract] |
| #45 | Metoprolol[Mesh] |
| #46 | Metoprolol [Title/Abstract] |
| #47 | Betaloc[Title/Abstract] |
| #48 | Lopresor[Title/Abstract] |
| #49 | Toprol [Title/Abstract] |
| #50 | Metoral[Title/Abstract] |
| #51 | Metolazone [Title/Abstract] |
| #52 | Nebivolol[Mesh] |
| #53 | Nebivolol[Title/Abstract] |
| #54 | Nebilet[Title/Abstract] |
| #55 | Bystolic[Title/Abstract] |
| #56 | Propranolol[Mesh] |
| #57 | Propranolol[Title/Abstract] |
| #58 | Inderal[Title/Abstract] |
| #59 | or/31-58 |
| #60 | Heart Diseases[Mesh] |
| #61 | Heart diseas*[Title/Abstract] |
| #62 | Cardiac diseas* [Title/Abstract] |
| #63 | Myocardial infarction[Title/Abstract] |
| #64 | Coronary disease[Title/Abstract] |
| #65 | Stroke[Title/Abstract] |
| #66 | Cardiotoxicity[Title/Abstract] |
| #67 | Cardiomyopathy[Title/Abstract] |
| #68 | Heart failure congestive[Title/Abstract] |
| #69 | Heart failure[Title/Abstract] |
| #70 | Cardiomyopathy congestive[Title/Abstract] |
| #71 | Ventricular dysfunction[Title/Abstract] |
| #72 | Ventricular dysfunction left[Title/Abstract] |
| #73 | Ventricular dysfunction[Title/Abstract] |
| #74 | Treatment[Title/Abstract] |
| #75 | Prevention[Title/Abstract] |
| #76 | Chemotherapy[Title/Abstract] |
| #77 | or/60-76 |
| #78 | Randomized controlled trial[Publication Type] |
| #79 | Controlled clinical trial[Publication Type] |
| #80 | Random*[Title/Abstract] |
| #81 | or/78-80 |
| #82 | #30 and #59 and #77 and #81 |
